# The effects of chemical and physical penetration enhancers on the percutaneous permeation of lidocaine through equine skin

**DOI:** 10.1186/1746-6148-10-138

**Published:** 2014-06-20

**Authors:** Jessica Stahl, Manfred Kietzmann

**Affiliations:** 1 Department of Pharmacology, Toxicology and Pharmacy, University of Veterinary Medicine Hannover, Foundation, Buenteweg 17, 30559, Hannover, Germany

**Keywords:** Lidocaine, Percutaneous Permeation, Topical application, Permeation enhancer, Microneedles, Equine skin

## Abstract

**Background:**

The effect of physical and chemical permeation enhancers on *in vitro* transdermal permeation of lidocaine was investigated in the horse.

Therefore, the effect of six vehicles (phosphate-buffered saline (PBS), 50% ethanol, 50% propylene glycol, 50% isopropylalcohol, 50% isopropylalcohol/isopropylmyristate and 50% dimethylsulfoxide) was examined as well as the effect of microneedle pretreatment with different needle lengths on transdermal drug delivery of lidocaine.

The skin was obtained from the thorax of six Warmblood horses and was stored up to two weeks at - 20°C. Franz-type diffusion cells were used to study the transdermal permeation through split skin (600 μm thickness). The amount of lidocaine in the receptor fluid was determined by UV–VIS high-performance liquid chromatography.

**Results:**

All investigated vehicle supplementations diminished the transdermal flux of lidocaine through equine skin in comparison to pure PBS except dimethylsulfoxide, which resulted in comparable permeation rates to PBS. The maximum flux (J_max_) was 1.6-1.8 fold lower for lidocaine applied in 50% ethanol, propylene glycol, isopropylalcohol and isopropylalcohol/isopropylmyristate. A significant higher J_max_ of lidocaine was observed when lidocaine was applied in PBS onto microneedle pretreated skin with similar permeation rates in both needle lengths. After 6 hours, 1.7 fold higher recovery rates were observed in the microneedle pretreated skin samples than in the untreated control samples. The lagtimes were reduced to 20–50% in the microneedle pretreated skin samples.

**Conclusion:**

Microneedles represent a promising tool for transdermal lidocaine application in the horse with a rapid systemic bioavailability.

## Background

Lidocaine (lignocaine) represents an amide local anesthetic drug which is also systemically used as an anti-arrhythmic drug [1]. It has recently also gained interest as systemically used prokinetic drug in the treatment of the postoperative ileus in horses [2-5].

Since the transdermal drug administration provides many advantages over oral treatments or injections, the approach of transdermal drug delivery systems has attracted the attention of many researchers all over the world not only in human medicine but also in veterinary research. A topically applied drug has to overcome the main skin barrier, the *stratum corneum*, which comprises dead corneocytes embedded in a lipid-rich domain mainly consisting of free fatty acids, cholesterol and ceramides [6]. Studies in humans have demonstrated the effectiveness of topically applied lidocaine for treating chronic, neuropathic, osteoarthritic and muscle related pain [7-12]. However, after placement of a single 5% lidocaine patch on each fore leg of horses, no lidocaine was detected in blood samples up to 12 hours of patch application [13]. Aside from this, after topical application of a single dose of 0.02% lidocaine cream to horses no lidocaine was detected in blood or urine samples, while it could be determined in urine samples of dogs in concentrations up to 48 ng/ml after multiple applications to canine skin [14].

The objective of the current work was, therefore, to assess the impact of chemical and mechanical permeation enhancers on lidocaine permeation through equine skin using a simple diffusion technique. First, five different chemical additives known as permeation enhancers of transdermal drug delivery (diemthylsulfoxide, ethanol, isopropylalcohol, isopropylmyristate and propylene glycol) were investigated for their impact on transdermal lidocaine delivery through equine skin. Second, physical penetration enhancement was performed using microneedles (200 μm and 300 μm long titanium needles) in order to pre-treat the equine skin samples before lidocaine application in the best aqueous solution determined in the first experimental setup.

## Method

### Chemicals

All chemicals used for the buffer solutions in the present study were of the highest purity available and were purchased from Merck, Darmstadt, Germany. Lidocaine hydrochloride monohydrate and dimethylsulfoxide were purchased from Merck as well. Ethanol was obtained from Applichem GmbH, Darmstadt, Germany. Propylene glycol was purchased from Riedel-de Haen, Seelze, Germany, isopropylalcohol from Lab-Scan, Dublin, Ireland, and isopropylmyristate from Sigma-Aldrich, Steinheim, Germany.

### Membranes

The skin was harvested from six male and female (3/3) Warmblood horses after euthanasia in the Clinic for Horses, University of Veterinary Medicine, Foundation, Hannover, for reasons not related to the present study. Thus, an ethical approval was not necessary for our study. The skin over the thoracic region was dissected away and was frozen at -20°C up to two weeks. The average age of the horses was 14 years (±3 years).

### *In vitro* permeation

The skin was defrosted at room temperature and the hair was removed with clippers before an electrical dermatome (Zimmer, Eschbach, Germany) was used to obtain 600 μm thick skin slices. Franz-type diffusion cells (6G-01-00-15-12, PermeGear, Riegelsville, PA, USA, and Gauer Glas, Püttlingen, Germany) with a diffusion area of 1.77 cm^2^ and a receptor volume of approximately 12 ml were filled with phosphate-buffered saline (PBS, pH 7.4; 1 l contains 0.2 g KCl, 8.0 g NaCl, 0.2 g KH_2_PO_4_, 1.44 g Na_2_HPO_4_ × 2H_2_O and deionised water) and were maintained at 34°C to provide 32°C skin samples. Small skin pieces (2 × 2 cm) were incubated in PBS for 30 minutes before mounting the skin samples with the *stratum corneum* side uppermost. After baseline samples (0.4 ml) were taken from the receptor chamber, 1 ml of each test substance solution was applied onto the skin. The permeation of lidocaine out of each test solution was examined in duplicate per animal, while six horses were investigated in total. Samples from the receptor chamber were taken at predefined times up to 6 hours or 22 hours.

### Experimental setup

To investigate the effect of different vehicles on lidocaine permeation, the following vehicles were used (2 mg/ml lidocaine): PBS, ethanol (50% in PBS w/w; EtOH), propylene glycol (50% in PBS w/w; PG), isopropylalcohol (50% in PBS w/w; IPA), isopropylalcohol/isopropylmyristate (50% in PBS w/w; IPM/IPA), and dimethylsulfoxide (50% in PBS w/w; DMSO). Furthermore, the effect of microneedle pretreatment on transdermal drug delivery of lidocaine was investigated using two microneedle rollers (Medik8, London, United Kingdom) with different needle lengths (200 μm and 300 μm), both of which possessed of 192 titanium needles in a cylindrical arrangement (diameter of 150 μm at the basis). The microneedle pretreatment was performed after an incubation phase in PBS for 30 minutes using skin samples which were placed on a styropor panel and fixed with needles beyond the subsequent diffusion area of the skin samples. The microneedle rollers were rolled in four axes radial [15] over the skin surface before 1 ml of the lidocaine solution in PBS was applied. Sample withdrawal from the receptor chamber (0.4 ml) was performed at predefined times with replacement by 0.4 ml PBS. The donor chambers were covered with parafilm (BRAND GmbH & CO KG, Wertheim, Germany).

### Analysis

The receptor medium samples were analysed by high-performance liquid chromatography utilizing a model 126 pump at 1 ml/min, a model 168 UV–VIS detector at 240 nm, and a model 507 autosampler (all Beckman, Fullerton, CA, USA) for the injection of 100 μl. A reversed phase HPLC column (LiChroCART 250–4, LiChrospher® 100 RP-18, 5 μm, Merck, Darmstadt, Germany) combined with a guard column (LiChroCART® 4–4 mm, LiChrospher®100 RP-18 (5 μm), Merck, Darmstadt, Germany) were used at 40°C. The mobile phase comprises 78% phosphate buffer (pH 5.5) and 20% acetonitrile, 1% triethylamine and 1% acetic acid degassed via sonication.

### Data analysis

An automated algorithm [16] was used to calculate the maximum flux (J_max_, μg/cm^2^/h) and the apparent permeability coefficient (P_app_, cm/h). Values from replicated *in vitro* experiments (2 replicates per treatment) at the same individual were used as average. Differences between diffusion parameters of control (PBS) and of the chemical additives/microneedle pretreated skin samples were evaluated using Kruskal-Wallis test followed by Dunnett´s multiple comparison test (GraphPad Prism 4.01 Software Inc., San Diego, USA). A 0.05 significance level was adopted.

## Results

**Figure 1:**
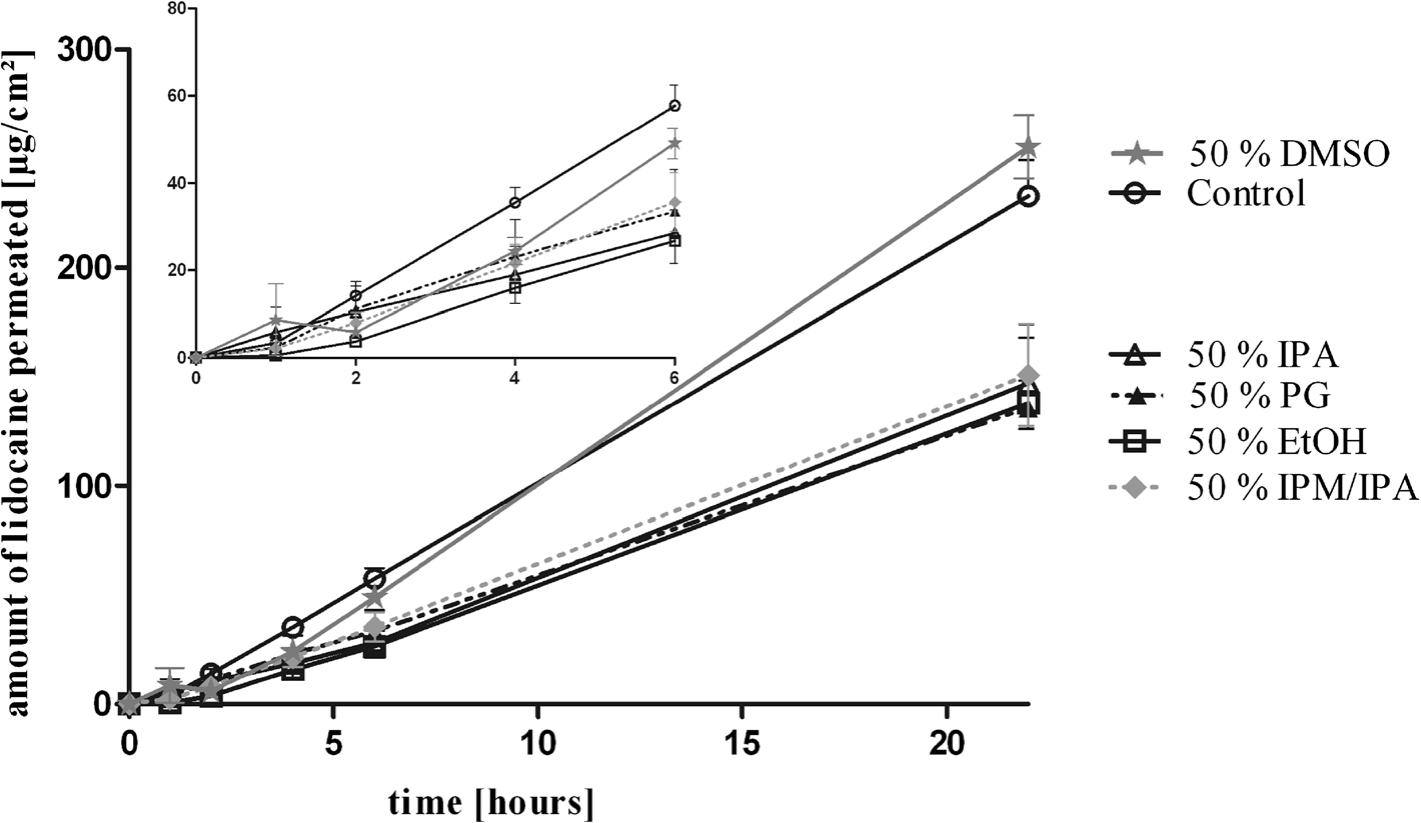
Lidocaine permeation of equine skin samples after application of topical formulations containing 2 mg/ml lidocaine (mean + SEM); * = p < 0.05; n = 6.

Lidocaine was able to permeate through equine skin out of all investigated formulations (Figure 1). The highest permeation of lidocaine was observed after administration of lidocaine in pure PBS and 50% DMSO. Formulations with 50% EtOH, PG, IPA and IPM/IPA diminished the permeation rate of lidocaine.

Parameters describing the features of the different vehicles on *in vitro* permeation of lidocaine are summarized in Table 1. Lidocaine in PBS showed the highest maximum flux J_max_ and the shortest lagtime through equine skin compared to EtOH, PG, IPA and IPM/IPA. Statistical significant differences were found for J_max_ and the apparent permeability coefficients of control vs. EtOH and PG (p < 0.05). The 6 hour-recoveries of lidocaine in 50% EtOH, PG and IPA (p < 0.01) and IPM/IPA (p < 0.05) were significantly different from the 6 hour-recovery of lidocaine in PBS (1.6 to 2.2-fold lower). The addition of 50% DMSO resulted in a comparable lidocaine permeation data to lidocaine applied in pure PBS. Although the lagtimes ranged from the mean of 0.9 hours (PBS) to 2.2 hours (IPA), no statistical significant differences were determined. There was no statistical significance between the different chemical additives.

**Table 1:** **Mean absorption parameters, J**_**max**_**, lagtime, P**_**app**_-value and recovery (mean ± standard deviation, STD), following application of different lidocaine formulations (PBS, EtOH (50% in PBS w/w), PG (50% in PBS w/w), IPA (50% in PBS w/w) , IPM/IPA (50% in PBS w/w), DMSO (50% in PBS w/w) containing 2 mg/ml lidocaine on equine skin; n = 6, * = p < 0.05, ** = p < 0.01

	**Vehicle**					
	**PBS**	**50% EtOH**	**50% PG**	**50% IPA**	**50% IPM/IPA(50/50 v/v)**	**50% DMSO**
	**Mean** **±** **STD**	**Mean** **±** **STD**	**Mean** **±** **STD**	**Mean** **±** **STD**	**Mean** **±** **STD**	**Mean** **±** **STD**
**J**_**max **_**(μg/cm**^**2**^**/h):**	**11.37** ± 1.81	**6.94** ± 4.53	**6.44** ± 1.43	**7.28** ± 3.00	**7.21** ± 2.72	**12.47** ± 1.66
**lagtime (h):**	**0.86** ± 1.03	**1.86** ± 1.99	**1.18** ± 0.95	**2.21** ± 1.21	**1.48** ± 0.61	**1.63** ± 0.53
**Papp (cm/s):**	**1.6E-06** ± 2.5E-07	**9.6E-07** ± 6.3E-07*	**8.9E-07** ± 2.0E-07*	**1.0E-06** ± 4.2E-07	**1.0E-06** ± 3.8E-07	**1.7E-06** ± 2.3E-07
**Recovery 6 h (%):**	**5.1** ± 1.0	**2.3** ± 1.5**	**2.9** ± 2.1**	**2.5** ± 1.5**	**3.1** ± 1.5*	**4.3** ± 0.5

*p < 0.05, **p < 0.01.

**Figure 2:**
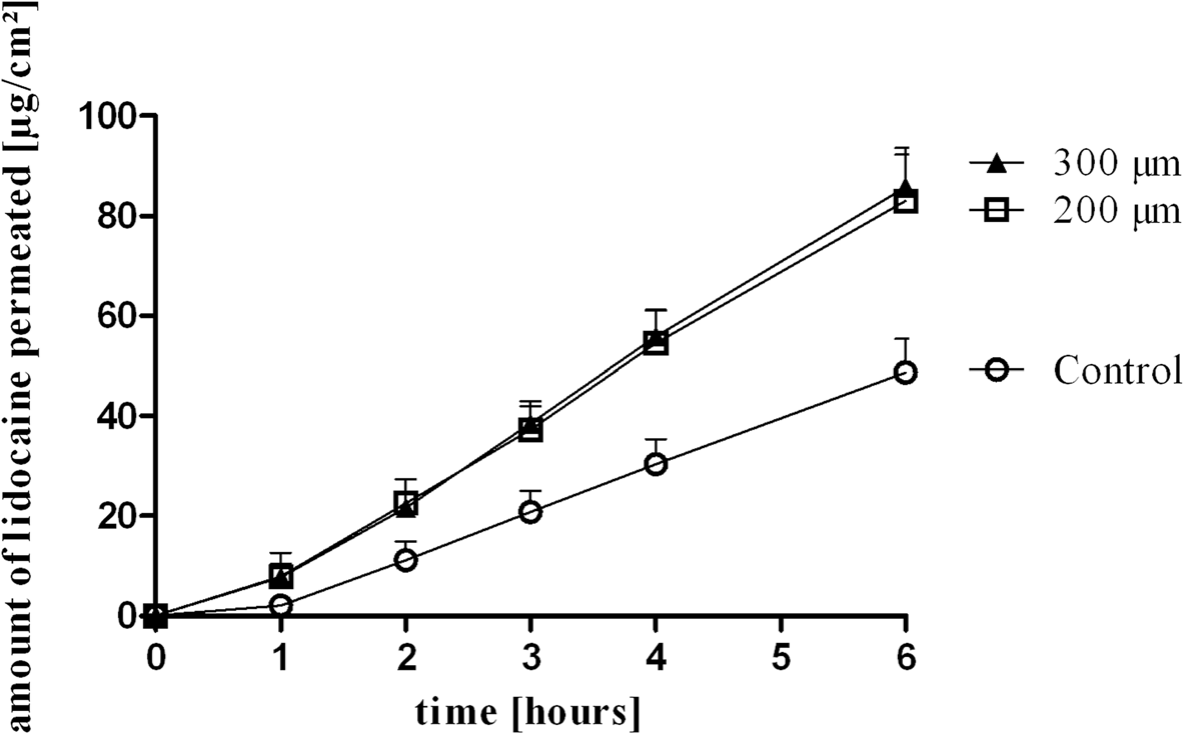
Lidocaine permeation of equine skin samples after application of 2 mg/ml lidocaine in PBS (mean + SEM) on microneedle pretreated skin samples; the microneedles were used with a needle length of 200 μm and 300 μm; * = p < 0.05; n = 6.

The microneedle pretreatment with both needle lengths significantly enhanced the permeation of lidocaine (Figure 2). Both needle lengths resulted in 1.4 higher permeation rates of lidocaine compared to control (p < 0.01) with diminished lagtimes. The lagtime was reduced from 1 hour in the untreated control skin to 0.8 hours in the 200 μm long microneedle treated skin samples and to 0.5 hours in the 300 μm long microneedle treated skin samples (Table 2). The 6-hour recoveries were approximately 1.7 fold higher in the microneedle pretreated skin samples (p < 0.05 (200 μm) and p < 0.01 (300 μm)).

**Table 2:** **Mean absorption parameters, J**_**max**_**, lagtime, P**_**app**_-value and recovery (mean ± standard deviation, SD), following application of 2 mg/ml lidocaine in PBS on microneedle pretreated equine skin; n = 6, * = p < 0.05, ** = p < 0.01, the microneedles were used with a needle length of 200 μm and 300 μm

	**Treatment**		
	**Control**	**200 μm**	**300 μm**
	**Mean** **±** **STD**	**Mean** **±** **STD**	**Mean** **±** **STD**
**J**_**max **_**(μg/cm**^**2**^**/h):**	**11.17** ± 4.63	**15.81** ± 3.35	**16.08** ± 3.93
**lagtime (h):**	**1.02** ± 0.70	**0.79** ± 0.28	**0.51** ± 0.34
**P**_**app **_**(cm/s):**	**1.6E-06** ± 6.4E-07	**2.2E-06** ± 4.7E-07**	**2.2E-06** ± 5.5E-07**
**Recovery 6 h (%):**	**4.3** ± 1.4	**7.3** ± 2.0*	**7.5** ± 1.7*

*p < 0.05, **p < 0.01.

## Discussion

Although there are many reports demonstrating that alcohols may act as skin permeability enhancers as solubilizing agents with delipidisation potential [17-20], in the present study no enhancing effect was observed for EtOH or IPA supplementation on transdermal permeation of lidocaine. Permeation of lidocaine, on the contrary, was diminished to alcohol supplementation to the vehicle compared to pure PBS which served as control. There are also various reports about permeation enhancements of sulfoxides like DMSO [19,21,22], which can increase lipid fluidity and promote drug partition [23,24] or polyols like PG [17,25,26], which are described to solvate α-keratin and occupy hydrogen bonding sites [27,28]. IPM exhibits a direct action on the *stratum corneum*, permeates into liposome bilayers and increases fluidity of membranes [19,29], but even this aliphatic fatty acid esters diminished lidocaine permeation through equine skin (53,54) comparable to PG. Only DMSO supplementation to the vehicle resulted in higher permeation rates of lidocaine compared to PG, IPA, EtOH or IPM/IPA, but similar permeation levels to pure PBS. Thus, the investigated chemicals show no effectiveness as permeation enhancers for lidocaine through equine skin, which may be due to the physicochemical nature of lidocaine. Furthermore, species react different to permeation enhancers and topically applied drugs, which has been shown e.g. for lidocaine patches in various species. 5% lidocaine patches administered to humans, cats and dogs resulted in measureable lidocaine concentrations in plasma of the examined species [30-33], while there was a lack of systemic absorption of lidocaine from 5 % patches placed on horses [13]. Furthermore, DMSO supplementation to the vehicle of transdermal applied lidocaine onto guinea pig skin resulted in higher transdermal permeation rates of lidocaine [34].

Interspecies differences in the success of penetration enhancement via chemical additives may be due to differences in skin morphology and biochemistry. Since the main skin barrier is maintained by the *stratum corneum*, its histology and lipid composition is of high importance. Concerning various reports about interspecies differences in these parameters [35-37], the lack of chemical penetration enhancement in the present study may be due to differences in the skin lipid composition of horses [38] compared with other species [37,39,40].

Due to the lack of chemical additives to enhance percutaneous permeation of lidocaine through equine skin, the second part of the present study was the investigation of the impact of mechanical permeation enhancers (microneedles) on lidocaine permeation through equine skin. Microneedles represent mechanical permeation enhancers in order to overcome the main skin barrier, the *stratum corneum*. This technology has been established to perforate the skin barrier without inducing pain or bleeding, as the needles are too short to stimulate the nerves and to damage blood vessels in the dermis [41,42]. Thus, the produced holes act as shunt ways for topically applied drugs with low permeability. In agreement with recent studies, microneedles enhance transdermal drug delivery of topically applied drugs [15,43-46]*.* After microneedle pretreatment, 1.7 fold higher amounts of lidocaine were determined in the present study in the receptor fluid after 6 hours with a 1.4 fold higher apparent permeability coefficient in the microneedle pretreated skin samples. Furthermore, the lag-times were reduced by 23% and 50% in dependence on the microneedle lengths. Consequently, from the present results it can be concluded that microneedle pretreatment results in a rapid onset of lidocaine action within the skin and in the organism, which is in accordance with recent studies about lidocaine-containing microneedle products with a rapid, safe, and prolonged local analgesic action [47,48].

## Conclusion

In conclusion, the results of the present study demonstrate significant differences in transdermal permeation of lidocaine dissolved in various vehicles and the high potential of microneedle pretreatment on transdermal permeation enhancement of lidocaine through equine skin.

### Clinical relevance

For the therapy of a postoperative ileus a plasma level of lidocaine of 1-2 μg/ml plasma is necessary [49]. Since the mean blood volume of a horse is 50 l [50], 50-100 μg lidocaine have to be in the organism in order to gain effective plasma levels. With the assumption of 7.5% recovery in the blood (recovery of 300 μm long microneedles in the acceptor chamber after 6 hours) a transdermal formulation containing 0.6-1.3 mg/ml may be adequate to reach effective plasma levels (when applied on microneedle perforated skin of the thorax). But therefore, several *in-vivo* studies in horses have to be performed.

## Abbreviations

(DMSO): Dimethylsulfoxide; (EtOH): Ethanol; (IPA): Isopropylalcohol; (IPM): Isopropylmyristate; (J_max_): Maximum flux; (PBS): Phosphate-buffered saline; (PG): Propylene glycol.

## Competing interests

The authors declare that they have no competing interest.

## Authors’ contributions

JS planned the study together with MK. JS coordinated the experiments, made the analysis and wrote the manuscript. Both authors have edited the manuscript. Both authors read and approved the final manuscript.
